# Molecular Beacon Assay Development for Severe Acute Respiratory Syndrome Coronavirus 2 Detection

**DOI:** 10.3390/s21217015

**Published:** 2021-10-22

**Authors:** Josué Carvalho, Jéssica Lopes-Nunes, Joana Figueiredo, Tiago Santos, André Miranda, Micaela Riscado, Fani Sousa, Ana Paula Duarte, Sílvia Socorro, Cândida Teixeira Tomaz, Mafalda Felgueiras, Rui Teixeira, Conceição Faria, Carla Cruz

**Affiliations:** 1 CICS-UBI—Centro de Investigação em Ciências da Saúde, Universidade da Beira Interior, Av. Infante D. Henrique, 6200-506 Covilhã, Portugal; josue.carvalho@fcsaude.ubi.pt (J.C.); jessica.nunes@ubi.pt (J.L.-N.); joana.figueiredo@ubi.pt (J.F.); tiago.santos@fcsaude.ubi.pt (T.S.); andre.miranda@ubi.pt (A.M.); micaela.riscado@ubi.pt (M.R.); fani.sousa@fcsaude.ubi.pt (F.S.); apduarte@fcsaude.ubi.pt (A.P.D.); ssocorro@fcsaude.ubi.pt (S.S.); ctomaz@ubi.pt (C.T.T.); 2 C4—Cloud Computing Competence Centre, UBIMedical, Universidade da Beira Interior, Estrada Municipal 506, 6200-284 Covilhã, Portugal; 3 Serviço de Patologia Clínica do Centro Hospitalar Universitário Cova da Beira (CHUCB), 6200-251 Covilhã, Portugal; mafalda.felgueiras@ulsm.min-saude.pt (M.F.); rteixeira@chcbeira.min-saude.pt (R.T.); cfaria@chcbeira.min-saude.pt (C.F.)

**Keywords:** RNA viruses, SARS-CoV-2, COVID-19, fluorescence detection, diagnosis

## Abstract

The fast spread of SARS-CoV-2 has led to a global pandemic, calling for fast and accurate assays to allow infection diagnosis and prevention of transmission. We aimed to develop a molecular beacon (MB)-based detection assay for SARS-CoV-2, designed to detect the ORF1ab and S genes, proposing a two-stage COVID-19 testing strategy. The novelty of this work lies in the design and optimization of two MBs for detection of SARS-CoV-2, namely, concentration, fluorescence plateaus of hybridization, reaction temperature and real-time results. We also identify putative G-quadruplex (G4) regions in the genome of SARS-CoV-2. A total of 458 nasopharyngeal and throat swab samples (426 positive and 32 negative) were tested with the MB assay and the fluorescence levels compared with the cycle threshold (Ct) values obtained from a commercial RT-PCR test in terms of test duration, sensitivity, and specificity. Our results show that the samples with higher fluorescence levels correspond to those with low Ct values, suggesting a correlation between viral load and increased MB fluorescence. The proposed assay represents a fast (total duration of 2 h 20 min including amplification and fluorescence reading stages) and simple way of detecting SARS-CoV-2 in clinical samples from the upper respiratory tract.

## 1. Introduction

Coronavirus disease 2019 (COVID-19) has instigated a global effort toward the development of fast and accurate detection techniques that would allow a more effective control of the global pandemic [1].

Reverse transcription-PCR (RT-PCR) is the mainstay of SARS-CoV-2 detection in which two or three target genes, mainly the open reading frame 1ab (ORF1ab), nucleocapsid protein (N), and envelope protein (E), are simultaneously amplified, according to WHO guidance [2]. These standard RT-PCR tests are expensive, time consuming, and their accuracy depends on the course of infection when the swab samples were collected [3]. Various alternative approaches are currently being developed to reduce the time for COVID-19 diagnosis [4,5], namely, loop-mediated isothermal amplification (LAMP), which works at a constant temperature and has already been used to successfully detect and identify viruses such as Zika [6]. However, it is a less accurate procedure, and only few samples can be run at a time in a low throughput setting [7]. CRISPR-Cas12/13-based SHERLOCK, DETECTR, CARVER, and PAC-MAN have also been applied to detect SARS-CoV-2 [7]. These tests are faster than RT-PCR tests (~30 min versus >1 h) because they use isothermal amplification technologies [7]. However, CRISPR diagnostics are less sensitive than the RT-PCR tests.

Therefore, the development of different approaches for faster and cheaper SARS-CoV-2 detection tests will be important to control the widespread transmission of COVID-19 [8,9].

MBs are target-specific oligonucleotides labeled with a fluorophore in one end (usually 5′-) and a quencher on the other (3′-end), which operate in an opposite way to TaqMan probes [8]. The MBs are designed to incorporate flanking complementary sequences on their 5′- and 3′-ends, and at low temperatures the ends anneal to form a hairpin, bringing the quencher and fluorophore into close proximity and quenching fluorescence [8]. For example, when SARS-CoV was spread in 2003, researchers developed a real-time RT-PCR assay using four MBs, targeting the S, E, M, and N genes to detect SARS-CoV and to distinguish between pathogenic and non-pathogenic strains [9]. Meanwhile, over the course of the COVID-19 pandemic, MBs paired with reverse transcription loop-mediated amplification to allow isothermal amplification from saliva to specifically detect the gene encoding the viral spike protein [10].

In this work, we designed two MBs to detect SARS-CoV-2 RNA from human samples. In particular, the MBs were designed to detect G-quadruplex (G4)-forming sequences within the SARS-CoV-2 genome. G4s are noncanonical secondary structures formed by guanine-rich nucleic acid sequences via Hoogsteen hydrogen bonding [11]. Recent studies have reported the presence of putative G4 sequences (PQS) in SARS-CoV-2 [12], and since PQS are usually highly conserved in pathogenic RNA virus genomes [13], they are attractive for detection approaches. The results cover the bioinformatic analysis of SARS-CoV-2 genome to design the MBs, structural characterization of the MBs, evaluation and optimization of the MBs using SARS-CoV-2 RNA extracts from 426 COVID-19 patients versus 32 controls, and a scalable fluorimetric analysis for the direct detection of SARS-CoV-2.

## 2. Materials and Methods

### 2.1. Bioinformatics Analysis of SARS-CoV-2 Genome

The SARS-CoV-2 reference genome sequence was obtained from the NCBI virus database (https://www.ncbi.nlm.nih.gov/labs/virus/vssi/#/ accessed on 2 April 2020) under the accession code NC_045512. PQS were systematically searched within the SARS-CoV-2 genome using the QGRS Mapper (http://bioinformatics.ramapo.edu/QGRS/analyze.php accessed on 30 November 2020) [14]. The default algorithm parameters were used as follows: max length of PQS was limited to 30; min G-tract length was set to 2; loop size was allowed to range from 0–10. PQS were scored based on their propensity to fold into G4 structures by the G-score. A total of 211,072 additional SARS-CoV-2 genome sequences isolated in several countries from 5 continents were obtained from the GISAID database. The pairwise alignment of SARS-CoV-2 sequences was performed using Multiple Alignment Fast Fourier Transform (MAFFT v7 server—https://mafft.cbrc.jp/alignment/server/ accessed on 13 October 2021) with default options. The consensus and conservation of PQS were visualized with Jalview v2.11.1.3 software.

Inclusivity/Specificity was evaluated by in silico analysis of published sequences using the assay primers and probes. BLASTn analysis was performed against publicly available nucleotide sequences found in the NCBI database. The Betacoronavirus Genbank database was queried and returned 56,747 SARS-CoV-2 whole genome sequences, updated as of 11 January 2021.

### 2.2. FRET Melting Assay

The experiments were performed on a CFX Connect™ Real-Time PCR Detection System (Bio-Rad, Hercules, CA, USA). Measurements were performed in PBS buffer (Sigma-Aldrich, St. Louis, MO, USA). The MBs were used at 0.2 µM. FAM fluorescence emission was recorded between 25–95 °C, with a temperature increment of 1 °C/min. The reverse melting was also obtained by monitoring FAM fluorescence emission during the cooling cycle between 95–25 °C. The excitation and detection wavelengths were 495 and 518 nm, respectively. Each experimental condition was tested in triplicate in at least two separate plates. The melting temperatures were determined from the normalized fluorescence curves as the temperature for which the normalized emission was 0.5.

#### 2.2.1. Circular Dichroism (CD) Spectroscopy

CD spectra were acquired in a Jasco J-815 spectrometer (Jasco, Easton, MD, USA), using a Peltier temperature controller (model CDF-426S/15). MB1 (5′-FAM-ACGCGCCCTTCGGAACCTTCTCCAACAACACCGCGCGT- BHQ1-3′) or MB2 (5′-FAM-ACGCGCCCAATACCATTAAACCTATAAGCCGCGCGT- BHQ1-3′) at 10 µM was dissolved in PBS (final volume of 250 µL).

Spectra were acquired through wavelengths ranging from 220 to 320 nm, with a scan speed of 100 nm/min, 1 nm bandwidth, 1 s integration time over 3 averaged accumulations in a quartz cuvette (Hellma, Jena, Germany) of 1 mm path length.

CD melting studies were performed in the temperature range 25–95 °C with a heating rate of 2 °C min^−1^ by monitoring the ellipticity at 270–275 nm. Spectra were acquired in the range of 0 to 1 molar equivalent of complementary sequence at 10 µM of MBs dissolved in PBS.

#### 2.2.2. Nuclear Magnetic Resonance (NMR) Spectroscopy

Standard zgesgp ^1^H NMR spectra were acquired on a 600 MHz Bruker Avance III spectrometer equipped with a QCI CryoProbe (proton frequency of 600 MHz). MBs were used at 50 μM in PBS 1× with 10% D_2_O (total volume of 180 μL) in a 3 mm NMR tube. MBs were titrated with complementary sequences at 50 μM. The experiments were therefore carried out at different temperatures (25 °C and *Tm* of MBs). During each experiment, samples were annealed by heating to 95 °C for 10 min followed by slow cool down to the target temperature.

For complementary sequences spectra acquisition, samples were diluted at 50 μM in PBS 1× with 10% D_2_O and supplemented with 150 mM KCl. The pre-saturation method was used for water suppression in 1D spectrum acquisition. All spectra were acquired and processed with the software Topspin 4.0.9. Chemical shifts (δ) were measured in ppm.

#### 2.2.3. Hybridization Temperature Determination

A total of 0.2 µM MB1 (5´-FAM-ACGCGCCCTTCGGAACCTTCTCCAACAACACCGCGCGT- BHQ1-3′) or MB2 (5´-FAM-ACGCGCCCAATACCATTAAACCTATAAGCCGCGCGT- BHQ1-3′) and 0.2 µM complementary sequences (for MB1: 5´-GGTGTTGTTGGAGAAGGTTCCGAAGG-3′; MB2: 5′-GGCTTATAGGTTTAATGGTATTGG-3′), in a final volume of 100 µL, were first incubated at 95 °C for 3 min, and different hybridization temperatures were then tested using a gradient mode ranging from 50.0 to 65.0 °C (65.0, 63.8, 62.0, 59.1, 55.7, 52.9, 51.0 and 50.0 °C) in a T100 Thermal Cycler (BIO-RAD, USA).

Later, the fluorescence levels were measured in a SpectraMax Gemini EM microplate reader (Molecular Devices, LLC., San Jose, CA, USA) with an excitation wavelength of 494 nm and an emission wavelength of 518 nm. The fluorescence levels were recorded at different time points (t = 0 min, t = 5 min, t = 21 min).

### 2.3. Clinical Samples

SARS-CoV-2 RNA was obtained from swab samples as part of routine care by the responsible clinical team of Centro Hospitalar Universitário Cova da Beira (CHUCB). No patient-identifiable data were accessible in this analysis. The need for written informed consent was waived by the Ethical board of CHUCB. The study was approved by the institutional ethics board of CHUCB (No.74/2020).

Nasopharyngeal and throat swab samples were collected from patients with suspicion of COVID-19 and used for extracting the SARS-CoV-2 RNA. After collection, the swabs were placed into a collection tube with 150 μL of virus preservation solution, and total RNA was extracted within 2 h using the NZY Viral RNA Isolation kit (NZYTech, Lisbon, Portugal).

#### 2.3.1. cDNA Synthesis and Amplification

cDNA synthesis and amplification were performed using the NZY First-Strand cDNA Synthesis kit, separate oligos (NZYTech; catalog number MB17002), and Speedy Supreme NZYTaq 2× Colourless Master Mix (NZYTech; catalog number MB39201), respectively. A 50 µL reaction was set up containing 5 µL of RNA sample, 1 µL Oligo(dT)18 primer mix (50 µM), 1 µL random hexamer mix (50 ng/ µL), 1 µL 10× Annealing Buffer, 10 µL NZYRT 2× Master Mix (no oligos), 2 µL NZYRT Enzyme Mix, 1.25 µL ORF1ab forward primer (5′-GCTAACATAGGTTGTAACCATA-3′), 1.25 µL ORF1ab reverse primer (5′-TTCAAGAAGGTTGTCATTAA-3′), 25 µL Speedy Supreme NZYTaq 2× Colourless Master Mix, and 2.5 µL nuclease-free water. Thermal cycling involved 65 °C for 5 min, 4 °C for 1 min, 25 °C for 5 min, 50 °C for 15 min, 85 °C for 5 min, and then 35 cycles of 95 °C for 5 min, 94 °C for 2 s, 50 °C for 5 s, and 72 °C for 2 s and final extension of 72 °C for 2 min. The total reaction time for cDNA synthesis and amplification was 1 h 23 m. Primers were designed and analyzed using Primer3web tool available online.

#### 2.3.2. cDNA Detection Using Molecular Beacon

To the resulting amplified cDNA was added 1 µM of MB1 (5′-FAM-ACGCGCCCTTCGGAACCTTCTCCAACAACACCGCGCGT- BHQ1-3′) and PBS up to 75 µL final volume. The mixture was then incubated for 10 min at 95 °C followed by 10 min at 52.9 °C in a T100 Thermal Cycler (BIO-RAD, USA). The fluorescence levels were then measured in a GloMax^®^ Promega microplate reader using 96-well black plates and a fluorescence blue filter with an excitation wavelength of 475 nm and an emission wavelength range of 500–550 nm.

### 2.4. Real-Time PCR for Clinical Samples

To detect the virus RNA-dependent RNA polymerase (RdRp) gene and to compare cycle threshold values (Ct-value) with the fluorescence levels obtained by our method, we performed RT-PCR in a CFX Connect™ Real-Time PCR Detection System (BIO-RAD, USA) using the SARS-CoV-2 One-Step RT-PCR kit, RUO (NZYTech; catalog number MD03191) following the manufacturer protocol. A 20 µL reaction was set up containing 8 µL of RNA sample, 10 µL of NZY speedy One-step RT-qPCR Master Mix, and 2 µL of SARS-CoV-2/RP primers/probe mix (FAM and JOE labeled, respectively). SARS-CoV-2/RP (8 × 10^5^ SARS-CoV-2 and RP copies) was used instead of the RNA template as a positive control and RNase/DNase free water was a negative control. Thermal cycling involved incubation at 50 °C for 20 min, followed by 95 °C for 2 min and then 40 cycles of 95 °C for 5 s and 60 °C for 30 s. In order to obtain an estimated end-point sensitivity of the assay, a standard curve was constructed by performing a dilution series of six 100 µL 1/10 dilutions of the provided SARS-CoV-2 copy number quantification (1 × 10^7^ copies/µL) in RNase/DNase free water.

## 3. Results

The work’s novelty is the combination of MBs with G4-forming sequences within the SARS-CoV-2 genome to increase sensitivity and speed of testing.

To identify putative PQS in the genome of SARS-CoV-2, the QGRS algorithm was employed to search G-rich sequences with general motif G_x_ N_y1_ G_x_ N_y2_ G_x_ N_y3_ G_x_ where x corresponds to the number of G-tetrads in the G4 structure and y1, y2 and y3 the loop length connecting the G-tetrads. PQS were scored based on their propensity to fold into G4 structures by the G-score. The algorithm was able to retrieve 16 overlapping PQS in the SARS-CoV-2 genome but only the PQS with G-score higher than 16 were considered (Table 1). These were found in important regulatory regions such as ORF1ab, spike protein (S), and nucleocapsid protein (N) coding regions. This suggests that G4 motifs may present regulatory roles in SARS-CoV-2 viral replication, assembly, and immune response modulation [12,15,16,17,18].

To analyze consensus and conservation of PQS, a total of 211,072 SARS-CoV-2 genome sequences from a broad geographic area, which included the most representative countries from 5 continents, were obtained from the GISAID database. The sequences were analyzed for the presence of the five PQS previously identified in the SARS-CoV-2 genome (Table 1). The results showed a high consensus and conservation of five of the highest-scoring sequences, with a conservation level above 98%. The sequence that belongs to the N region revealed 90.82% of conservation in all sequences (Table 1).

Taking into account the combined bioinformatic data, two sequences were selected as targets for the development of MBs to detect SARS-CoV-2 RNA based on the G-score, conservation, and consensus for the MBs design. The selected sequences were 5′-GGTGTTGTTGGAGAAGGTTCCGAAGG-3′ and 5′-GGCTTATAGGTTTAATGGTATTGG-3′, which belong to the ORF1ab and S regions, respectively. The consensus and conservation of the two selected sequences were then analyzed among the different variants, including new emerging variants of SARS-CoV-2 (Table 2). Both sequences are highly conserved among all SARS-CoV-2 variants with a global conservation level of more than 97.77%. To gain insight into the consensus and conservation of PQS in each country and variant, we performed a more detailed analysis of each country. The results are highlighted in Table S1 (Supplementary Materials) and showed a high level of consensus and conservation in variants from different countries, except the Delta variant in Portugal. Interestingly, the results revealed that only approximately 75% of the analyzed sequences contained the target sequence from ORF1ab. Considering these results, we performed a deep sequence analysis of the alignment data and found a frequent mutation from cytosine to thymine in position 20 (Nucleotide underlined) of sequence 5′-GGTGTTGTTGGAGAAGGTTCCGAAGG-3′. However, the mutation occurred in the loop region of PQS and did not inhibit G4 formation.

Both sequences were already shown to form parallel-stranded G4 structures in vitro in potassium-buffered conditions [12] and were therefore suitable targets for viral detection as reported for other viral diseases [19].

Two MBs with sequence complementarity to each of the previously selected sequences were then designed and characterized. The MBs contained the complementary sequence of the identified PQS (loop region), extended by 5′ and 3′ complementary sequences (stem region) to form a hairpin-like structure, and a fluorophore-quencher pair, specifically, 5′-FAM and BHQ1-3′. The in silico analysis showed that the MBs demonstrated homology with all available SARS-CoV-2 genomes in the NCBI Betacoronavirus database as of January 2021 (100% identity), and significant homology (≥80%) to SARS coronavirus, pangolin coronavirus, and bat SARS-like/SARS-related coronavirus (Table S2). The specificity of the assay was provided by both the MBs and the primers used to amplify the target regions of the MBs. While the MBs presented ≥80% homology with other related CoVs, the primers did not show any significant alignment, and therefore the specificity of the assay was ensured. These organisms were further analyzed in silico and as they did not have nearby or correctly oriented primers or MBs with significant alignment (>80%) to bi-directionally amplify out a PCR product that could be detected by the proposed method, no potential cross-reactivity was expected. Furthermore, no significant alignments were found with other organisms, particularly after selecting respiratory pathogens such as Influenza A and B, Rhinovirus, *Legionella pneumophilia,* and *Mycobacterium tuberculosis* (a list of organisms tested can be found in Table S2). Combining the designed sets of primers and MBs, there were no significant homologies with the human genome, other coronaviruses, or human microflora that would predict potential false positive results using the proposed assay. Altogether, the results from the in silico analysis suggested that no cross-reactivity (false positive) could be expected by using the MBs with the following sequences: MB1: 5′-FAM-ACGCG**CCCTTCGGAACCTTCTCCAACAACACC**GCGCGT-BHQ1-3′ and MB2: 5′-FAM-ACGCG**CCCAATACCATTAAACCTATAAGCC**GCGCGT-BHQ1-3′, for ORF1ab and S protein targets, respectively. Letters in bold represent the complementary sequence of the target sequences.

**Figure 1:**
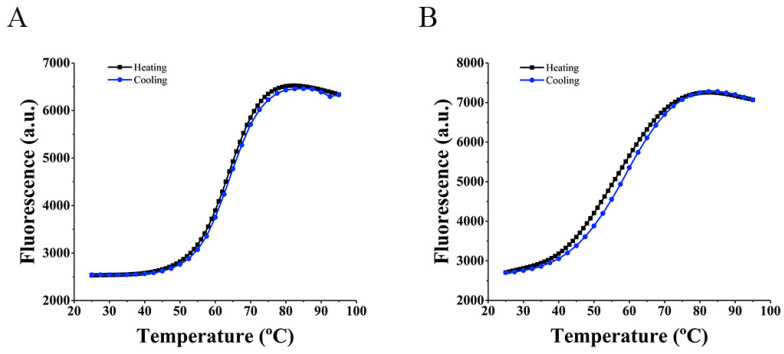
Thermal stabilization of (**A**) MB1 and (**B**) MB2 measured by FRET melting assay.

The ability of the MBs to undergo conformational rearrangements allowing target detection, as well as the melting temperature, were determined by FRET melting assay (Figure 1). As suggested by the FRET melting, both probes melted around 60 °C (63.1 and 55.3 °C for MB1 and MB2, respectively).

**Figure 2:**
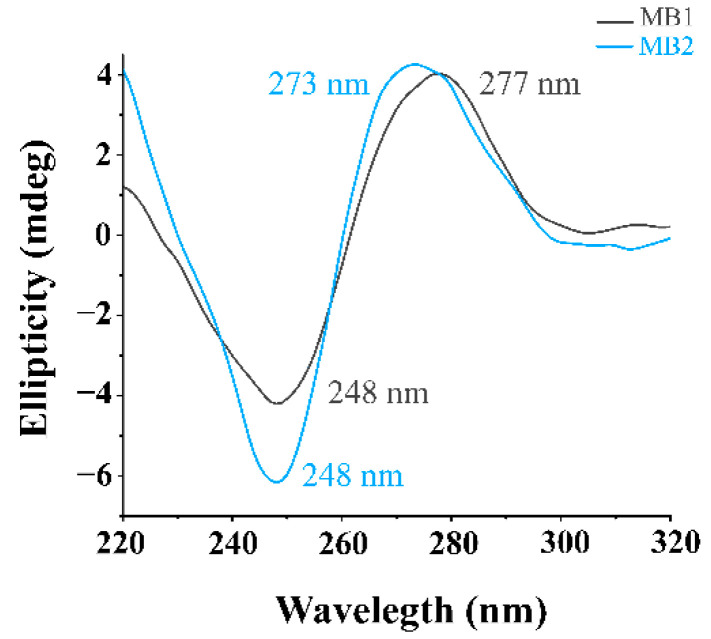
CD spectra of MB1 and MB2 acquired in PBS.

The structural characterization of the MBs was assessed by CD spectroscopy. The MBs spectra showed a negative band at 248 nm and a positive band at 277 and 273 nm for MB1 and MB2, respectively, indicating the presence of B-DNA form in both MBs (Figure 2).

Moreover, the melting curves of MB1 and MB2 in the presence of the complementary sequences were performed by CD melting (Figure S2) and the T_m_ were 73.3 and 63.1 °C, respectively.

**Figure 3:**
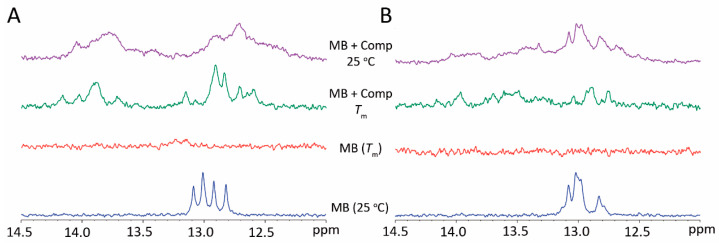
^1^H NMR spectra (imino region) of (**A**) MB1 and (**B**) MB2 in PBS 1× at 25 °C (blue spectra), at the beacon’s melting temperature (middle spectra, with and without the complementary sequence in green and red, respectively) and in the presence of the complementary sequences at 25 °C (purple spectra).

The ^1^H NMR spectra of MBs (bottom spectra) showed the existence of imino signals around 13 ppm (Figure 3), characteristic of Watson–Crick GC base pairing [20]. This result was indicative of the formation of a hairpin structure by base pairing of the flanking complementary sequences [21]. Upon denaturation of both MBs (middle spectra) and addition of the MBs’ complementary sequences followed by cooling to room temperature, a set of imino signals indicative of target hybridization was observed around 13 and 14 ppm, for GC and AT base pairing, respectively.

After the structural characterization of MBs, the hybridization temperature was determined in a thermocycler. For that purpose, complementary sequences were added to the probes. The mixture was fully denatured at 95 °C and then different temperatures (65.0, 63.8, 62.0, 59.1, 55.7, 52.9, 51.0 and 50.0 °C) were tested to select the hybridization temperature. After that, the fluorescence levels of the mixtures were measured to identify which temperature promoted the highest fluorescence intensity and thus the optimal target hybridization conditions.

As observed in Table 3, the hybridization temperatures that resulted in an improvement of the fluorescence intensity were 52.9 and 59.1 °C for MB1 and MB2, respectively, indicating these were the most efficient for MBs hybridization.

Additionally, the fluorescence intensity was measured at different time points (t = 0 min, t = 5 min, t = 21 min). Based on the results seen in Table 3 the optimal time point to measure the fluorescence was immediately after hybridization of the MBs with the complementary sequences, since the fluorescence tends to decrease over time.

The MBs were then used to detect SARS-CoV-2 RNA in clinical samples isolated from the upper respiratory tract by naso- and oropharyngeal swabs. A total of 426 samples with confirmed SARS-CoV-2 detection by RT-PCR analysis were provided by the CHUCB and used to optimize fluorescence detection. All samples in the control group (32) were confirmed as negative by RT-PCR.

cDNA was first synthesized and amplified in order to achieve a better sensitivity of detection. cDNA synthesis and amplification protocols were optimized in terms of time and reaction yield. The optimal reaction time was evaluated to be 1 h 23 m. Regarding MB2, which targeted a guanine-rich sequence within the spike (S) protein-coding region, despite its ability to function as a detection probe, its applicability in the proposed protocol was hindered. This was due to the fact that no amplification of the target S sequence was observed by end-point PCR in any of the SARS-CoV-2 positive samples tested. As a rule of thumb, PCR primers generally range in length from 15–30 bases, and should contain 40–60% GC content, a T_m_ above 52–58 °C, and care should be taken to avoid sequences that might produce internal secondary structure. In the case of the S target sequence, the designed primers presented a moderate propensity to form dimers and internal secondary structures such as hairpins. This, together with the fact that the melting temperature of the primers was closer to the annealing temperature set (50 °C) when compared to that of ORF1ab primers, could explain why no amplification product was observed on agarose gel electrophoresis. Therefore, the following studies were only performed with MB1. The amplified cDNA was then incubated with MB1 (in PBS for 10 min at 95 °C and 10 min at 52.9 °C) in a thermocycler and the fluorescence was measured in a microplate reader using a fluorescence blue filter with an excitation wavelength of 475 nm and an emission wavelength range of 500–550 nm.

**Figure 4:**
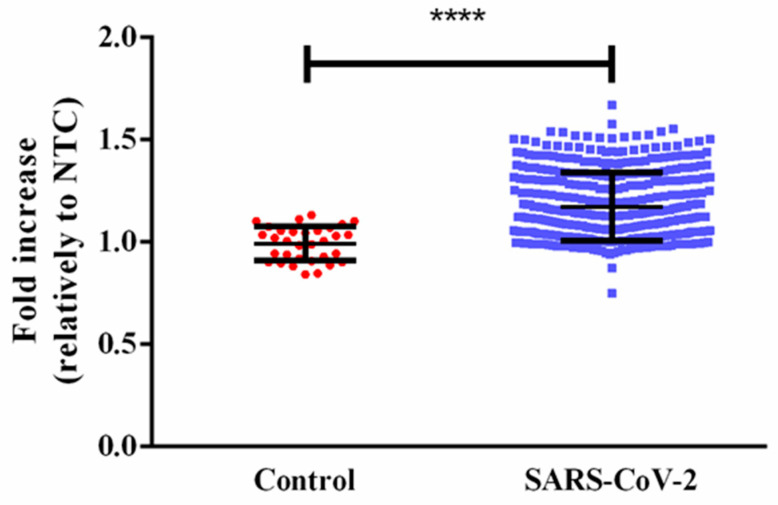
Fold increase of fluorescence in SARS-CoV-2 positive human samples relative to the no template control (NTC). **** *p* < 0.0001.

The obtained fluorescence levels were normalized relative to the no template control (NTC), and the results are presented in Figure 4 in terms of fluorescence fold increase. The results showed a statistically significant difference between the controls and the positive SARS-CoV-2 human samples (mean fluorescence for control and SARS-CoV-2 positive samples, respectively).

**Figure 5:**
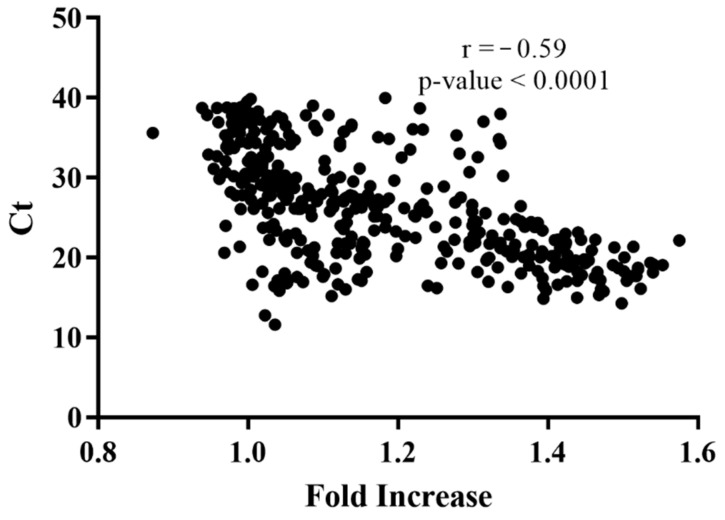
Correlation between cycle threshold (Ct) amplification of SARS-CoV-2 in human samples and fluorescence fold increase relative to the no template control (NTC).

The proof of concept of the MB assay was performed by comparing the fluorescence fold increase with the Ct values of SARS-CoV-2 amplification in the RT-PCR analysis of the clinical samples. The results are presented in Figure 5. A moderate, negative, but statistically significant correlation existed between fluorescence fold increase and Ct values when values from both sources were combined (Pearson’s r = −0.59; *p*-value < 0.0001). The test can reliably detect samples where Ct < 39. The fold increase in fluorescence intensity was inversely proportional to the Ct values obtained with the commercial SARS-CoV-2 RT-PCR test, which detects the virus RdRp gene (Figure 5) that is part of the ORF1ab [22] and equally targeted by MB1; and it therefore allows reliable comparability of detection methods. The analytical performance characteristics with 95% confidence intervals were calculated for sensitivity and specificity of the MB method when compared to the RT-PCR results. Considering only the samples with Ct < 30, i.e., those that could be accurately diagnosed with the method described in this manuscript, we were able to obtain a sensitivity of 96.3% (95% CI, 93.3–98.2%) and a specificity of 46.9% (95% CI, 29.1–65.3%) as shown in the two-by-two analysis. The sensitivity was slightly higher than that of RT-PCR methods and significantly higher than rapid antigen tests [3,4].

## 4. Discussion

We described an MB-based assay for the detection of SARS-CoV-2 in clinical samples.

The work’s novelty is the design and optimization of MBs to detect the G4 regions of SARS-CoV-2 RNA. Despite real-time RT-PCR (RT-qPCR) being considered the “gold standard” for the detection of SARS-CoV-2 RNA, the routine diagnosis by RT-qPCR is still a limitation for many laboratories, mainly because of organization incapability and/or the disproportionate relationship between demand and supply of inputs, especially in less developed countries and regions. The current requirement for inputs, reagents/consumables, and equipment and the incapacity of the supplier companies to satisfy the demand represents an additional obstacle to the timely diagnosis of COVID-19. In this context, and to increase the diagnostic capability for COVID-19, we aimed at developing a sensitive and specific protocol for the detection of SARS-CoV-2 through end-point RT-PCR as a potential alternative for situations in which RT-qPCR is not possible and/or available. The proposed method offers several advantages such as:

i. Ease of result interpretation, requiring less training and enabling health care staff to use the test correctly. The current RT-qPCR tests employ multiplex reactions that render a multitude of amplification plots for two to four different genes, which are not readily interpreted by health care staff with limited training in molecular biology techniques or molecular diagnosis. Furthermore, despite OMS recommendations, the cycle threshold (Ct) and copy number values provided by RT-qPCR are not being used to drive the routine clinical practice. Therefore, the extra unused information provided by RT-qPCR is creating a needless and expensive bottleneck. End-point RT-PCR provides the same Positive/Negative result for the presence/absence of SARS-CoV-2 RNA at a potentially lower cost and higher throughput, with easier interpretation.
ii. Less expensive lab apparatus and reagents needed. The proposed test does not require expensive real-time thermocyclers, being possible to execute using a simple end-point thermocycler or programmable heating block, and a plate reader or fluorescence reading apparatus. This enables the laboratories of developing countries with poor access to heavy lab machinery to implement the method in their facilities using existent equipment as end-point PCR is already used to diagnose other infectious diseases such as Hepatitis B, Ebola, and HIV. Furthermore, as the reactions are performed using a single set of primers and a single molecular beacon, the users can easily implement the protocol using existent enzyme master mixes and reagents, without the need to purchase expensive RT-qPCR SARS-CoV-2 detection kits.

The method comprised two MBs to detect the SARS-CoV-2 ORF1ab and S genes. The MBs were designed based on the complementary sequences from SARS-CoV-2 RNA identified by the QGRS algorithm with the highest G-score, conservation, and consensus. The presence of the confirmed G4-forming sequences and the candidate’s frequency, uniqueness, and conservation rates among all the different SARS-CoV-2 clinical samples tested in silico, rendered the G4 regions suitable targets for the development of viral detection methods.

The conservation levels of the selected target regions belonging to ORF1ab and S regions were approximately 98.07 and 99.97%, respectively. Furthermore, the level of conservation was preserved at new emergent variants, suggesting the applicability of the molecular beacons in SARS-CoV-2 detection.

It was demonstrated that despite the high variability of viral genomes, G4s with important functional roles can be expected to be conserved among strains and isolates belonging to the same viral species, which seemed to be the case for SARS-CoV-2 based on recent reports [12,15,16,17,18]. Altogether these finding pointed to the suitability of targeting G4 regions to diagnose SARS-CoV-2 infection. We included in 5′- and 3′-ends a stem region to form a hairpin-like structure and a fluorophore-quencher pair, 5´-FAM and BHQ1-3′, respectively. The MBs exhibited a melting temperature above 63.1 and 55.3 °C for MB1 and MB2, respectively, which was suitable for hybridization detection assays. Normalized fluorescence signals for MBs and MB-target hybrids were also determined to give thermal denaturation profiles (Table 3). In the 52.9–60 °C temperature range, the MB-target hybrids elicited significantly stronger fluorescence than the probe alone, allowing clear detection of the target sequences. The temperatures of 52.9 and 59.1 °C for MB1 and MB2, respectively, were chosen as optimal for fluorescence detection, in which upon recognition of the target, the fluorophore and the quencher would be spatially separated owing to hybridization, resulting in FAM fluorescence emission. The optimal concentration for MBs was found to be 0.2 µM. The structural characterization of MBs performed by CD and NMR spectroscopies corroborated the formation of the hairpin-like structure and the ability to hybridize with their targets. The MB-based SARS-CoV-2 detection assay was optimized to be completed within 2 h 20 min, including cDNA synthesis, amplification, and MB incubation in a thermocycler to identify the viral sequence by fluorescence emission.

The applicability of MB2 in the proposed assay was hindered because no amplification of the target S sequence was observed by end-point PCR in any of the SARS-CoV-2 positive samples tested. This might be due to the inefficiency of the primers to hybridize with the target region and/or the formation of stable G4 structures (Figure S1) that inhibit Taq polymerase. Indeed, this same principle was used as an assay to study G4 binders entitled PCR-stop assay [23].

Finally, we also compared the Ct values obtained with a commercial RT-PCR kit for amplification of SARS-CoV-2 in 426 positive samples with our SARS-CoV-2 MB detection assay. The results presented in Figure S3 demonstrated a moderate correlation between viral load (given by the Ct values) and MB fluorescence emission, suggesting that our assay was reliable and could also be used to detect SARS-CoV-2 samples with a great level of sensitivity. The MB assay was able to detect and discriminate the positive samples from the negative controls, as demonstrated by the plot of the fold increase of fluorescence in SARS-CoV-2 human positive samples relative to the NTC presented in Figure 5. There were numerous SARS-CoV-2 positive samples whose fluorescence fell in the same range of controls; however, these samples presented Ct values above 30 and it has been suggested that patients with a lower viral load are most likely not infectious [24,25,26]. Nevertheless, the method could reliably detect SARS-CoV-2 positive samples with Ct < 39, which correlated with clinical significance. The sensitivity of the MB method was 96.3% and the specificity 46.9%. The sensitivity was similar to the current methods while the specificity was lower than that reported for reference RT-PCR methods [3,4]. Nevertheless, the method was accurate at detecting true positive samples and did not give rise to false negatives that are of greater clinical relevance.

Altogether, our findings highlight the need to investigate new methods for SARS-CoV-2 detection based on MBs that can be translated to field testing and clinical settings with no access to heavy lab machinery.

## Figures and Tables

**Table 1:** SARS-CoV-2 genome sequences with the highest PQS and G-scores were obtained from patients from 23 countries from 5 continents.

Genome Region	Position	Length	QGRS ^1^	G-Score	Total of Genomes Analyzed	Genomes with PQS	Conservation Level (%)
ORF1ab	1,574	26	GGTGTTGTTGGAGAAGGTTCCGAAGG	19	211,072	207,006	98.07
	13,385	20	GGTATGTGGAAAGGTTATGG	18		210,888	99.91
S	24,215	20	GGTTGGACCTTTGGTGCAGG	17	211,072	210,803	99.87
	24,268	24	GGCTTATAGGTTTAATGGTATTGG	19		210,999	99.97
	25,197	22	GGCCATGGTACATTTGGCTAGG	17		207,837	98.47
N	28,903	15	GGCTGGCAATGGCGG	18	211,072	191,696	90.82

^1^ G-tracts are underlined.

**Table 2:** Analysis of the two sequences selected as targets for the development of MBs in SARS-CoV-2 variants.

Variant	SARS-CoV-2 Gene	SARS-CoV-2 G4 Sequence	Total of Sequences Analyzed	Number of Sequences with PQS	Conservation (%)
Alpha	ORF1ab	GGTGTTGTTGGAGAAGGTTCCGAAGG	153,813	151,813	99.02
	S	GGCTTATAGGTTTAATGGTATTGG		153,259	99.96
Delta	ORF1ab	GGTGTTGTTGGAGAAGGTTCCGAAGG	184,757	180,642	97.77
	S	GGCTTATAGGTTTAATGGTATTGG		184,691	99.96
Beta	ORF1ab	GGTGTTGTTGGAGAAGGTTCCGAAGG	13,185	12,942	98.16
	S	GGCTTATAGGTTTAATGGTATTGG		13,175	99.92
Gamma	ORF1ab	GGTGTTGTTGGAGAAGGTTCCGAAGG	36,027	35,930	99.73
	S	GGCTTATAGGTTTAATGGTATTGG		36,012	99.96
Lambda	ORF1ab	GGTGTTGTTGGAGAAGGTTCCGAAGG	211	211	100
	S	GGCTTATAGGTTTAATGGTATTGG		211	100
Mu	ORF1ab	GGTGTTGTTGGAGAAGGTTCCGAAGG	3,266	3,247	99.42
	S	GGCTTATAGGTTTAATGGTATTGG		3,263	99.91

**Table 3:** Fluorescence intensity of MB1 and MB2 after hybridization with the complementary sequences at different temperatures and time points.

	MB1			MB2		
Temperature (°C)	t = 0 Min	t = 5 Min	t = 21 Min	t = 0 Min	t = 5 Min	t = 21 Min
65	51.05	45.09	42.31	61.29	66.81	65.94
63.8	58.65	57.64	56.51	60.09	62.01	60.86
62	59.68	57.81	56.75	78.69	72.64	64.07
59.1	64.42	63.26	62.18	82.31	73.63	65.91
55.7	59.58	57.75	55.69	49.42	48.48	45.86
52.9	72.69	68.76	63.37	77.06	68.49	62.01
51	68.03	66.23	61.45	76.58	69.99	66.78
50	60.36	62.92	60.34	70.81	65.35	60.38

## Data Availability

The (Not applicable.) GISAID EpiCoV™ Database was used for download SARS-CoV-2 genome sequences (https://www.gisaid.org/ accessed on 13 October 2021).

## Supplementary Materials

The following are available online at https://www.mdpi.com/article/10.3390/s21217015/s1, Figure S1: ^1^H NMR spectra (imino region) of MB1 and MB2 complementary sequences at 25 °C, Figure S2: Melting Curves Of MB1 and MB2 in presence of complementary sequences C1 and C2, respectively, Figure S3: Correlation between cycle threshold (Ct) amplification of SARS-CoV-2 in human samples, fluorescence fold increase relative to the no template control (NTC) and samples with different amounts of viral RNA. Table S1: Conservation analysis of PQS in 23 countries and 6 variants of SARS-CoV-2, Table S2: In silico analysis for primers and MBs targeting ORF1ab and S regions.
