# Supplementary material for: Molecular Beacon Assay Development for Severe Acute Respiratory Syndrome Coronavirus 2 Detection
Source: Sensors (Basel). 2021 Oct 22;21(21):7015. doi: 10.3390/s21217015 (PMC8587319; doi:10.3390/s21217015)
Supplement: Supplementary file 1 [file sensors-21-07015-s001.zip › sensors-1359119-supplementary.pdf]

Supplementary Material

Molecular beacon assay development for Severe Acute Respiratory Syndrome Coronavirus 2 detection

Josué Carvalho [1], Jéssica Lopes-Nunes [1], Joana Figueiredo [1], Tiago Santos [1], André Miranda [1], Micaela Riscado [1], Fani Sousa [1,2], Ana Paula Duarte [1,2], Sílvia Socorro [1], Cândida Tomaz [1,2], Mafalda Felgueiras [3], Rui Teixeira [3], Conceição Faria [3], Carla Cruz [1]*

[1]CICS-UBI—Centro de Investigação em Ciências da Saúde, Universidade da Beira Interior, Av. Infante D. Henrique, 6200-506 Covilhã , Portugal; josue.carvalho@fcsaude.ubi.pt (J.C.); jessi-ca.nunes@ubi.pt (J.L.-N.); joana.figueiredo@ubi.pt (J.F.); tiago.santos@fcsaude.ubi.pt (T.S.); andre.miranda@ubi.pt (A.M.); micaela.riscado@ubi.pt (M.R.); fani.sousa@fcsaude.ubi.pt (F.S.); apduarte@fcsaude.ubi.pt (A.P.D.); ssocorro@fcsaude.ubi.pt (S.S.); ctomaz@ubi.pt (C.T.T.)

[2]C4—Cloud Computing Competence Centre, UBIMedical, Universidade da Beira Interior, EM506, 6200-284 Covilhã, Portugal

[3]Serviço de Patologia Clínica do Centro Hospitalar Universitário Cova da Beira (CHUCB), 6200-251 Covilhã, Portugal;

mafalda.felgueiras@ulsm.min-saude.pt (M.F.); rteixeira@chcbeira.min-saude.pt (R.T.); cfa-ria@chcbeira.min-saude.pt (C.F.)

*Correspondence: carlacruz@fcsaude.ubi.pt

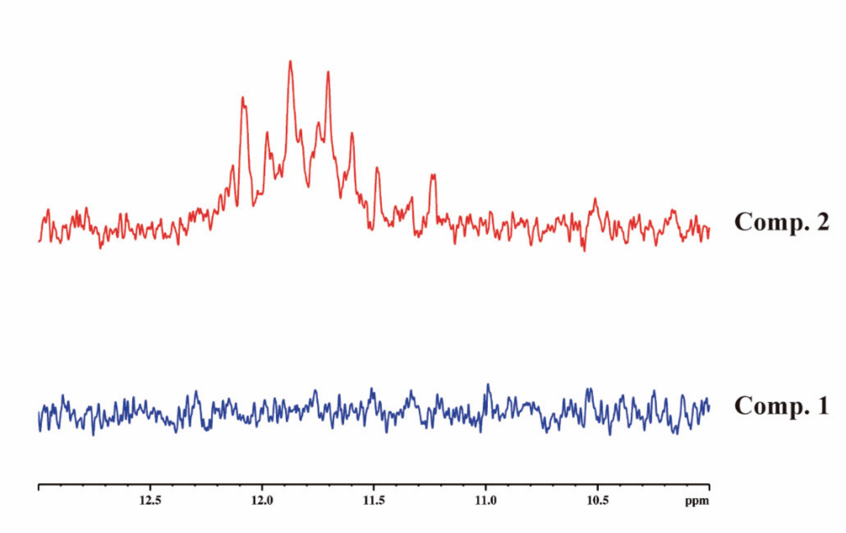

Figure S1. ^{1}H NMR spectra (imino region) of MB1 and MB2 complementary sequences at 25 °C.

Table S1. Analysis of the conservation of PQS in 23 countries and 6 variants of SARS-CoV-2.

Country	Variant	Gene	QGRS	Total of sequences	Number of sequences with PQS	Conservation (%)
South Korea	alpha	ORF1	GGTGTTGTTGGAGAAGGTTCCGAAGG	728	714	98.08
		S	GGCTTATAGGTTTAATGGTATTGG		728	100
	Delta	ORF1	GGTGTTGTTGGAGAAGGTTCCGAAGG	4306	4306	100
		S	GGCTTATAGGTTTAATGGTATTGG		4304	99.95
	Beta	ORF1	GGTGTTGTTGGAGAAGGTTCCGAAGG	36	35	97.22
		S	GGCTTATAGGTTTAATGGTATTGG		36	100
	Gamma	ORF1	GGTGTTGTTGGAGAAGGTTCCGAAGG	14	14	100
		S	GGCTTATAGGTTTAATGGTATTGG		14	100
	Lambda	ORF1	GGTGTTGTTGGAGAAGGTTCCGAAGG	0	0	0
		S	GGCTTATAGGTTTAATGGTATTGG		0	0
	Mu	ORF1	GGTGTTGTTGGAGAAGGTTCCGAAGG	0	0	0
		S	GGCTTATAGGTTTAATGGTATTGG		0	0
Denmark	alpha	ORF1	GGTGTTGTTGGAGAAGGTTCCGAAGG	10000	9849	98.49
		S	GGCTTATAGGTTTAATGGTATTGG		9997	99.97
	Delta	ORF1	GGTGTTGTTGGAGAAGGTTCCGAAGG	10000	9952	99.52
		S	GGCTTATAGGTTTAATGGTATTGG		10000	100
	Beta	ORF1	GGTGTTGTTGGAGAAGGTTCCGAAGG	106	97	91.51
		S	GGCTTATAGGTTTAATGGTATTGG		106	100
	Gamma	ORF1	GGTGTTGTTGGAGAAGGTTCCGAAGG	34	34	100
		S	GGCTTATAGGTTTAATGGTATTGG		34	100
	Lambda	ORF1	GGTGTTGTTGGAGAAGGTTCCGAAGG	0	0	0
		S	GGCTTATAGGTTTAATGGTATTGG		0	0
	Mu	ORF1	GGTGTTGTTGGAGAAGGTTCCGAAGG	4	4	100
		S	GGCTTATAGGTTTAATGGTATTGG		4	100
Japan	alpha	ORF1	GGTGTTGTTGGAGAAGGTTCCGAAGG	10000	9953	99.53

		S	GGCTTATAGGTTTAATGGTATTGG		10000	100
	Delta	ORF1	GGTGTTGTTGGAGAAGGTTCCGAAGG	10000	9989	9989
		S	GGCTTATAGGTTTAATGGTATTGG		10000	100
	Beta	ORF1	GGTGTTGTTGGAGAAGGTTCCGAAGG	112	111	99.11
		S	GGCTTATAGGTTTAATGGTATTGG		112	100
	Gamma	ORF1	GGTGTTGTTGGAGAAGGTTCCGAAGG	124	124	100
		S	GGCTTATAGGTTTAATGGTATTGG		124	100
	Lambda	ORF1	GGTGTTGTTGGAGAAGGTTCCGAAGG	0	0	0
		S	GGCTTATAGGTTTAATGGTATTGG		0	0
	Mu	ORF1	GGTGTTGTTGGAGAAGGTTCCGAAGG	5	5	100
		S	GGCTTATAGGTTTAATGGTATTGG		5	100
**Sweden**	alpha	ORF1	GGTGTTGTTGGAGAAGGTTCCGAAGG	10000	9991	99.91
		S	GGCTTATAGGTTTAATGGTATTGG		9998	99.98
	Delta	ORF1	GGTGTTGTTGGAGAAGGTTCCGAAGG	10000	9851	98.51
		S	GGCTTATAGGTTTAATGGTATTGG		9993	99.93
	Beta	ORF1	GGTGTTGTTGGAGAAGGTTCCGAAGG	2173	2141	98.53
		S	GGCTTATAGGTTTAATGGTATTGG		2173	100
	Gamma	ORF1	GGTGTTGTTGGAGAAGGTTCCGAAGG	132	132	100
		S	GGCTTATAGGTTTAATGGTATTGG		132	100
	Lambda	ORF1	GGTGTTGTTGGAGAAGGTTCCGAAGG	0	0	0
		S	GGCTTATAGGTTTAATGGTATTGG		0	0
	Mu	ORF1	GGTGTTGTTGGAGAAGGTTCCGAAGG	0	0	0
		S	GGCTTATAGGTTTAATGGTATTGG		0	0
**Italy**	alpha	ORF1	GGTGTTGTTGGAGAAGGTTCCGAAGG	10000	9980	99.80
		S	GGCTTATAGGTTTAATGGTATTGG		9997	99.97
	Delta	ORF1	GGTGTTGTTGGAGAAGGTTCCGAAGG	10000	9873	98.73
		S	GGCTTATAGGTTTAATGGTATTGG		10000	100
	Beta	ORF1	GGTGTTGTTGGAGAAGGTTCCGAAGG	99	88	88.89
		S	GGCTTATAGGTTTAATGGTATTGG		99	100
	Gamma	ORF1	GGTGTTGTTGGAGAAGGTTCCGAAGG	2080	2076	99.81
		S	GGCTTATAGGTTTAATGGTATTGG		2080	100
	Lambda	ORF1	GGTGTTGTTGGAGAAGGTTCCGAAGG	3	3	100
		S	GGCTTATAGGTTTAATGGTATTGG		3	100
	Mu	ORF1	GGTGTTGTTGGAGAAGGTTCCGAAGG	61	59	96.72
		S	GGCTTATAGGTTTAATGGTATTGG		61	100
**Portugal**	alpha	ORF1	GGTGTTGTTGGAGAAGGTTCCGAAGG	3885	3836	98.74
		S	GGCTTATAGGTTTAATGGTATTGG		3884	99.97
	Delta	ORF1	GGTGTTGTTGGAGAAGGTTCCGAAGG	7014	5305	75.63
		S	GGCTTATAGGTTTAATGGTATTGG		7007	99.90
	Beta	ORF1	GGTGTTGTTGGAGAAGGTTCCGAAGG	80	80	100
		S	GGCTTATAGGTTTAATGGTATTGG		80	100
	Gamma	ORF1	GGTGTTGTTGGAGAAGGTTCCGAAGG	137	137	100
		S	GGCTTATAGGTTTAATGGTATTGG		137	100
	Lambda	ORF1	GGTGTTGTTGGAGAAGGTTCCGAAGG	1	1	100
		S	GGCTTATAGGTTTAATGGTATTGG		1	100
	Mu	ORF1	GGTGTTGTTGGAGAAGGTTCCGAAGG	21	20	95.24

		S	GGCTTATAGGTTTAATGGTATTGG		21	100
**United Kingdom**	alpha	ORF1	GGTGTTGTTGGAGAAGGTTCCGAAGG	10000	9964	99.64
		S	GGCTTATAGGTTTAATGGTATTGG		9993	99.93
	Delta	ORF1	GGTGTTGTTGGAGAAGGTTCCGAAGG	10000	9955	99.55
		S	GGCTTATAGGTTTAATGGTATTGG		9996	99.96
	Beta	ORF1	GGTGTTGTTGGAGAAGGTTCCGAAGG	792	788	99.49
		S	GGCTTATAGGTTTAATGGTATTGG		787	99.37
	Gamma	ORF1	GGTGTTGTTGGAGAAGGTTCCGAAGG	168	167	99.40
		S	GGCTTATAGGTTTAATGGTATTGG		168	100
	Lambda	ORF1	GGTGTTGTTGGAGAAGGTTCCGAAGG	1	1	100
		S	GGCTTATAGGTTTAATGGTATTGG		1	100
	Mu	ORF1	GGTGTTGTTGGAGAAGGTTCCGAAGG	45	42	93.33
		S	GGCTTATAGGTTTAATGGTATTGG		45	100
**USA**	alpha	ORF1	GGTGTTGTTGGAGAAGGTTCCGAAGG	10000	9927	99.27
		S	GGCTTATAGGTTTAATGGTATTGG		9993	99.93
	Delta	ORF1	GGTGTTGTTGGAGAAGGTTCCGAAGG	10000	9972	99.72
		S	GGCTTATAGGTTTAATGGTATTGG		9993	99.93
	Beta	ORF1	GGTGTTGTTGGAGAAGGTTCCGAAGG	1908	1846	96.75
		S	GGCTTATAGGTTTAATGGTATTGG		1907	99.95
	Gamma	ORF1	GGTGTTGTTGGAGAAGGTTCCGAAGG	10000	9977	99.77
		S	GGCTTATAGGTTTAATGGTATTGG		9997	99.97
	Lambda	ORF1	GGTGTTGTTGGAGAAGGTTCCGAAGG	83	83	100
		S	GGCTTATAGGTTTAATGGTATTGG		83	100
	Mu	ORF1	GGTGTTGTTGGAGAAGGTTCCGAAGG	2469	2456	99.47
		S	GGCTTATAGGTTTAATGGTATTGG		2467	99.92
**Australia**	alpha	ORF1	GGTGTTGTTGGAGAAGGTTCCGAAGG	401	395	98.50
		S	GGCTTATAGGTTTAATGGTATTGG		401	100
	Delta	ORF1	GGTGTTGTTGGAGAAGGTTCCGAAGG	7966	6984	87.67
		S	GGCTTATAGGTTTAATGGTATTGG		7966	100
	Beta	ORF1	GGTGTTGTTGGAGAAGGTTCCGAAGG	50	50	100
		S	GGCTTATAGGTTTAATGGTATTGG		50	100
	Gamma	ORF1	GGTGTTGTTGGAGAAGGTTCCGAAGG	3	3	100
		S	GGCTTATAGGTTTAATGGTATTGG		3	100
	Lambda	ORF1	GGTGTTGTTGGAGAAGGTTCCGAAGG	1	1	100
		S	GGCTTATAGGTTTAATGGTATTGG		1	100
	Mu	ORF1	GGTGTTGTTGGAGAAGGTTCCGAAGG	0	0	0
		S	GGCTTATAGGTTTAATGGTATTGG		0	0
**Spain**	alpha	ORF1	GGTGTTGTTGGAGAAGGTTCCGAAGG	10000	9861	98.61
		S	GGCTTATAGGTTTAATGGTATTGG		9995	99.95
	Delta	ORF1	GGTGTTGTTGGAGAAGGTTCCGAAGG	10000	9777	97.77
		S	GGCTTATAGGTTTAATGGTATTGG		9998	99.98
	Beta	ORF1	GGTGTTGTTGGAGAAGGTTCCGAAGG	255	255	100
		S	GGCTTATAGGTTTAATGGTATTGG		255	100
	Gamma	ORF1	GGTGTTGTTGGAGAAGGTTCCGAAGG	901	901	100
		S	GGCTTATAGGTTTAATGGTATTGG		901	100
	Lambda	ORF1	GGTGTTGTTGGAGAAGGTTCCGAAGG	90	90	100

		S	GGCTTATAGGTTTAATGGTATTGG		90	100
	Mu	ORF1	GGTGTTGTTGGAGAAGGTTCCGAAGG	483	483	100
		S	GGCTTATAGGTTTAATGGTATTGG		482	99.79
**India**	alpha	ORF1	GGTGTTGTTGGAGAAGGTTCCGAAGG	4939	4888	98.97
		S	GGCTTATAGGTTTAATGGTATTGG		4934	99.90
	Delta	ORF1	GGTGTTGTTGGAGAAGGTTCCGAAGG	10000	9922	99.22
		S	GGCTTATAGGTTTAATGGTATTGG		9988	99.88
	Beta	ORF1	GGTGTTGTTGGAGAAGGTTCCGAAGG	115	114	99.13
		S	GGCTTATAGGTTTAATGGTATTGG		115	100
	Gamma	ORF1	GGTGTTGTTGGAGAAGGTTCCGAAGG	1	1	100
		S	GGCTTATAGGTTTAATGGTATTGG		1	100
	Lambda	ORF1	GGTGTTGTTGGAGAAGGTTCCGAAGG	0	0	0
		S	GGCTTATAGGTTTAATGGTATTGG		0	0
	Mu	ORF1	GGTGTTGTTGGAGAAGGTTCCGAAGG	0	0	0
		S	GGCTTATAGGTTTAATGGTATTGG		0	0
**Netherlands**	alpha	ORF1	GGTGTTGTTGGAGAAGGTTCCGAAGG	10000	9849	98.49
		S	GGCTTATAGGTTTAATGGTATTGG		9998	99.98
	Delta	ORF1	GGTGTTGTTGGAGAAGGTTCCGAAGG	10000	9931	99.31
		S	GGCTTATAGGTTTAATGGTATTGG		10000	100
	Beta	ORF1	GGTGTTGTTGGAGAAGGTTCCGAAGG	631	602	95.40
		S	GGCTTATAGGTTTAATGGTATTGG		631	100
	Gamma	ORF1	GGTGTTGTTGGAGAAGGTTCCGAAGG	523	517	98.85
		S	GGCTTATAGGTTTAATGGTATTGG		523	100
	Lambda	ORF1	GGTGTTGTTGGAGAAGGTTCCGAAGG	2	2	100
		S	GGCTTATAGGTTTAATGGTATTGG		2	100
	Mu	ORF1	GGTGTTGTTGGAGAAGGTTCCGAAGG	63	63	100
		S	GGCTTATAGGTTTAATGGTATTGG		63	100
**Canada**	alpha	ORF1	GGTGTTGTTGGAGAAGGTTCCGAAGG	10000	9948	99.48
		S	GGCTTATAGGTTTAATGGTATTGG		9994	99.94
	Delta	ORF1	GGTGTTGTTGGAGAAGGTTCCGAAGG	10000	9773	97.73
		S	GGCTTATAGGTTTAATGGTATTGG		10000	100
	Beta	ORF1	GGTGTTGTTGGAGAAGGTTCCGAAGG	776	770	99.23
		S	GGCTTATAGGTTTAATGGTATTGG		776	100
	Gamma	ORF1	GGTGTTGTTGGAGAAGGTTCCGAAGG	10000	9991	99.91
		S	GGCTTATAGGTTTAATGGTATTGG		9993	99.93
	Lambda	ORF1	GGTGTTGTTGGAGAAGGTTCCGAAGG	14	14	100
		S	GGCTTATAGGTTTAATGGTATTGG		14	100
	Mu	ORF1	GGTGTTGTTGGAGAAGGTTCCGAAGG	20	20	100
		S	GGCTTATAGGTTTAATGGTATTGG		20	100
**Switzerland**	alpha	ORF1	GGTGTTGTTGGAGAAGGTTCCGAAGG	10000	9753	97.53
		S	GGCTTATAGGTTTAATGGTATTGG		9998	99.98
	Delta	ORF1	GGTGTTGTTGGAGAAGGTTCCGAAGG	10000	9914	99.14
		S	GGCTTATAGGTTTAATGGTATTGG		9993	99.93
	Beta	ORF1	GGTGTTGTTGGAGAAGGTTCCGAAGG	256	256	100
		S	GGCTTATAGGTTTAATGGTATTGG		256	100
	Gamma	ORF1	GGTGTTGTTGGAGAAGGTTCCGAAGG	183	183	100

		S	GGCTTATAGGTTTAATGGTATTGG		183	100
	Lambda	ORF1	GGTGTTGTTGGAGAAGGTTCCGAAGG	6	6	100
		S	GGCTTATAGGTTTAATGGTATTGG		6	100
	Mu	ORF1	GGTGTTGTTGGAGAAGGTTCCGAAGG	36	36	100
		S	GGCTTATAGGTTTAATGGTATTGG		36	100
**South Africa**	alpha	ORF1	GGTGTTGTTGGAGAAGGTTCCGAAGG	145	145	100
		S	GGCTTATAGGTTTAATGGTATTGG		145	100
	Delta	ORF1	GGTGTTGTTGGAGAAGGTTCCGAAGG	3508	3470	98.92
		S	GGCTTATAGGTTTAATGGTATTGG		3507	99.97
	Beta	ORF1	GGTGTTGTTGGAGAAGGTTCCGAAGG	2627	2555	97.26
		S	GGCTTATAGGTTTAATGGTATTGG		2625	99.92
	Gamma	ORF1	GGTGTTGTTGGAGAAGGTTCCGAAGG	0	0	0
		S	GGCTTATAGGTTTAATGGTATTGG		0	0
	Lambda	ORF1	GGTGTTGTTGGAGAAGGTTCCGAAGG	0	0	0
		S	GGCTTATAGGTTTAATGGTATTGG		0	0
	Mu	ORF1	GGTGTTGTTGGAGAAGGTTCCGAAGG	0	0	0
		S	GGCTTATAGGTTTAATGGTATTGG		0	0
**Belgium**	alpha	ORF1	GGTGTTGTTGGAGAAGGTTCCGAAGG	17005	16719	98.32
		S	GGCTTATAGGTTTAATGGTATTGG		16998	99.96
	Delta	ORF1	GGTGTTGTTGGAGAAGGTTCCGAAGG	11819	11729	99.24
		S	GGCTTATAGGTTTAATGGTATTGG		11814	99.96
	Beta	ORF1	GGTGTTGTTGGAGAAGGTTCCGAAGG	832	832	100
		S	GGCTTATAGGTTTAATGGTATTGG		832	100
	Gamma	ORF1	GGTGTTGTTGGAGAAGGTTCCGAAGG	1203	1200	99.75
		S	GGCTTATAGGTTTAATGGTATTGG		1203	100
	Lambda	ORF1	GGTGTTGTTGGAGAAGGTTCCGAAGG	5	5	100
		S	GGCTTATAGGTTTAATGGTATTGG		5	100
	Mu	ORF1	GGTGTTGTTGGAGAAGGTTCCGAAGG	32	32	100
		S	GGCTTATAGGTTTAATGGTATTGG		32	100
**Ireland**	alpha	ORF1	GGTGTTGTTGGAGAAGGTTCCGAAGG	14905	14865	99.73
		S	GGCTTATAGGTTTAATGGTATTGG		14902	99.98
	Delta	ORF1	GGTGTTGTTGGAGAAGGTTCCGAAGG	13754	13663	99.34
		S	GGCTTATAGGTTTAATGGTATTGG		13752	99.99
	Beta	ORF1	GGTGTTGTTGGAGAAGGTTCCGAAGG	40	40	100
		S	GGCTTATAGGTTTAATGGTATTGG		40	100
	Gamma	ORF1	GGTGTTGTTGGAGAAGGTTCCGAAGG	18	18	100
		S	GGCTTATAGGTTTAATGGTATTGG		18	100
	Lambda	ORF1	GGTGTTGTTGGAGAAGGTTCCGAAGG	0	0	0
		S	GGCTTATAGGTTTAATGGTATTGG		0	0
	Mu	ORF1	GGTGTTGTTGGAGAAGGTTCCGAAGG	2	2	100
		S	GGCTTATAGGTTTAATGGTATTGG		2	100
**Singapore**	alpha	ORF1	GGTGTTGTTGGAGAAGGTTCCGAAGG	175	173	98.86
		S	GGCTTATAGGTTTAATGGTATTGG		175	100
	Delta	ORF1	GGTGTTGTTGGAGAAGGTTCCGAAGG	5647	5628	99.66
		S	GGCTTATAGGTTTAATGGTATTGG		5640	99.88
	Beta	ORF1	GGTGTTGTTGGAGAAGGTTCCGAAGG	195	194	99.49

		S	GGCTTATAGGTTTAATGGTATTGG		194	99.49
	Gamma	ORF1	GGTGTTGTTGGAGAAGGTTCCGAAGG	8	8	100
		S	GGCTTATAGGTTTAATGGTATTGG		8	100
	Lambda	ORF1	GGTGTTGTTGGAGAAGGTTCCGAAGG	0	0	0
		S	GGCTTATAGGTTTAATGGTATTGG		0	0
	Mu	ORF1	GGTGTTGTTGGAGAAGGTTCCGAAGG	0	0	0
		S	GGCTTATAGGTTTAATGGTATTGG		0	0
**China**	alpha	ORF1	GGTGTTGTTGGAGAAGGTTCCGAAGG	14	14	100
		S	GGCTTATAGGTTTAATGGTATTGG		14	100
	Delta	ORF1	GGTGTTGTTGGAGAAGGTTCCGAAGG	84	84	100
		S	GGCTTATAGGTTTAATGGTATTGG		84	100
	Beta	ORF1	GGTGTTGTTGGAGAAGGTTCCGAAGG	2	2	100
		S	GGCTTATAGGTTTAATGGTATTGG		2	100
	Gamma	ORF1	GGTGTTGTTGGAGAAGGTTCCGAAGG	0	0	0
		S	GGCTTATAGGTTTAATGGTATTGG		0	0
	Lambda	ORF1	GGTGTTGTTGGAGAAGGTTCCGAAGG	0	0	0
		S	GGCTTATAGGTTTAATGGTATTGG		0	0
	Mu	ORF1	GGTGTTGTTGGAGAAGGTTCCGAAGG	0	0	0
		S	GGCTTATAGGTTTAATGGTATTGG		0	0
**Saudi Arabia**	alpha	ORF1	GGTGTTGTTGGAGAAGGTTCCGAAGG	4	4	100
		S	GGCTTATAGGTTTAATGGTATTGG		4	100
	Delta	ORF1	GGTGTTGTTGGAGAAGGTTCCGAAGG	0	0	0
		S	GGCTTATAGGTTTAATGGTATTGG		0	0
	Beta	ORF1	GGTGTTGTTGGAGAAGGTTCCGAAGG	1	1	100
		S	GGCTTATAGGTTTAATGGTATTGG		1	100
	Gamma	ORF1	GGTGTTGTTGGAGAAGGTTCCGAAGG	0	0	0
		S	GGCTTATAGGTTTAATGGTATTGG		0	0
	Lambda	ORF1	GGTGTTGTTGGAGAAGGTTCCGAAGG	0	0	0
		S	GGCTTATAGGTTTAATGGTATTGG		0	0
	Mu	ORF1	GGTGTTGTTGGAGAAGGTTCCGAAGG	0	0	0
		S	GGCTTATAGGTTTAATGGTATTGG		0	0
**France**	alpha	ORF1	GGTGTTGTTGGAGAAGGTTCCGAAGG	10000	9896	98.96
		S	GGCTTATAGGTTTAATGGTATTGG		9993	99.93
	Delta	ORF1	GGTGTTGTTGGAGAAGGTTCCGAAGG	10000	9944	99.44
		S	GGCTTATAGGTTTAATGGTATTGG		9998	99.98
	Beta	ORF1	GGTGTTGTTGGAGAAGGTTCCGAAGG	2092	2078	99.33
		S	GGCTTATAGGTTTAATGGTATTGG		2091	99.95
	Gamma	ORF1	GGTGTTGTTGGAGAAGGTTCCGAAGG	484	482	99.59
		S	GGCTTATAGGTTTAATGGTATTGG		484	100
	Lambda	ORF1	GGTGTTGTTGGAGAAGGTTCCGAAGG	1	1	100
		S	GGCTTATAGGTTTAATGGTATTGG		1	100
	Mu	ORF1	GGTGTTGTTGGAGAAGGTTCCGAAGG	15	15	100
		S	GGCTTATAGGTTTAATGGTATTGG		15	100
**Iceland**	alpha	ORF1	GGTGTTGTTGGAGAAGGTTCCGAAGG	538	538	100
		S	GGCTTATAGGTTTAATGGTATTGG		537	99.81
	Delta	ORF1	GGTGTTGTTGGAGAAGGTTCCGAAGG	3383	3364	99.44

		S	GGCTTATAGGTTTAATGGTATTGG		3383	100
	Beta	ORF1	GGTGTTGTTGGAGAAGGTTCCGAAGG	1	1	100
		S	GGCTTATAGGTTTAATGGTATTGG		1	100
	Gamma	ORF1	GGTGTTGTTGGAGAAGGTTCCGAAGG	14	14	100
		S	GGCTTATAGGTTTAATGGTATTGG		14	100
	Lambda	ORF1	GGTGTTGTTGGAGAAGGTTCCGAAGG	0	0	0
		S	GGCTTATAGGTTTAATGGTATTGG		0	0
	Mu	ORF1	GGTGTTGTTGGAGAAGGTTCCGAAGG	0	0	0
		S	GGCTTATAGGTTTAATGGTATTGG		0	0
**Brazil**	alpha	ORF1	GGTGTTGTTGGAGAAGGTTCCGAAGG	581	551	94.84
		S	GGCTTATAGGTTTAATGGTATTGG		581	100
	Delta	ORF1	GGTGTTGTTGGAGAAGGTTCCGAAGG	7276	7256	99.73
		S	GGCTTATAGGTTTAATGGTATTGG		7275	99.99
	Beta	ORF1	GGTGTTGTTGGAGAAGGTTCCGAAGG	6	6	100
		S	GGCTTATAGGTTTAATGGTATTGG		6	100
	Gamma	ORF1	GGTGTTGTTGGAGAAGGTTCCGAAGG	10000	9951	99.51
		S	GGCTTATAGGTTTAATGGTATTGG		9995	99.95
	Lambda	ORF1	GGTGTTGTTGGAGAAGGTTCCGAAGG	4	4	100
		S	GGCTTATAGGTTTAATGGTATTGG		4	100
	Mu	ORF1	GGTGTTGTTGGAGAAGGTTCCGAAGG	10	10	100
		S	GGCTTATAGGTTTAATGGTATTGG		10	100

Table S2. ***In silico*** **analysis for primers and MBs targeting ORF1ab and S regions.**

		In silico analysis for % homology/identity					
		ORF 1ab			S		
Microorganism	Genbank Accession Code	Forward Primer	Reverse Primer	MB	Forward Primer	Reverse Primer	MB
Coronavirus 229E	NC_002645.1	No alignment was found			No alignment was found		
Coronavirus OC43	NC_006213.1	No alignment was found			No alignment was found		
Coronavirus HKU-1	NC_006577.2	No alignment was found			No alignment was found		
Coronavirus NL63	NC_005831.2	No alignment was found			No alignment was found		
SARS-coronavirus	NC_004718.3	No alignment was found			No alignment was found		
MERS-coronavirus	NC_019843.3	No alignment was found			No alignment was found		
Human adenovirus 2	AC_000007.1	No alignment was found			No alignment was found		
Human adenovirus 5	AC_000008.1	No alignment was found			No alignment was found		
Human adenovirus 54	NC_012959.1	No alignment was found			No alignment was found		
Human adenovirus A	NC_001460.1	No alignment was			No alignment was		

		found	found
Human adenovirus B1	NC_011203.1	No alignment was found	No alignment was found
Human adenovirus B2	NC_011202.1	No alignment was found	No alignment was found
Human adenovirus C	NC_001405.1	No alignment was found	No alignment was found
Human adenovirus D	NC_010956.1	No alignment was found	No alignment was found
Human adenovirus E	NC_003266.2	No alignment was found	No alignment was found
Human adenovirus F	NC_001454.1	No alignment was found	No alignment was found
Human adenovirus type 1	AC_000017.1	No alignment was found	No alignment was found
Human adenovirus type 35	AC_000019.1	No alignment was found	No alignment was found
Human adenovirus type 7	AC_000018.1	No alignment was found	No alignment was found
Human metapneumovirus	NC_039199.1	No alignment was found	No alignment was found
Human parainfluenza virus 1	NC_003461.1	No alignment was found	No alignment was found
Human parainfluenza virus 2	NC_003443.1	No alignment was found	No alignment was found
Human parainfluenza virus 3	NC_001796.2	No alignment was found	No alignment was found
Human parainfluenza virus4a	NC_021928.1	No alignment was found	No alignment was found
Influenza A virus H1N1	GCF_000865725	No alignment was found	No alignment was found
Influenza A virus H3N2	GCF_000865085	No alignment was found	No alignment was found
Influenza A virus H5N1	GCF_000864105	No alignment was found	No alignment was found
Influenza A virus H7N9	GCF_000928555	No alignment was found	No alignment was found
Influenza B virus	GCF_000820495	No alignment was found	No alignment was found
Influenza C virus	GCF_000856665.10	No alignment was found	No alignment was found
Enterovirus (e.g. EV68)	NC_038308.1	No alignment was found	No alignment was found
Human rhinovirus 1	NC_038311.1	No alignment was found	No alignment was found
Human rhinovirus 3	NC_038312.1	No alignment was found	No alignment was found
Human rhinovirus 14	NC_001490.1	No alignment was found	No alignment was found

Human rhinovirus 89	NC_001617.1	No alignment was found			No alignment was found		
Human rhinovirus C	NC_009996.1	No alignment was found			No alignment was found		
Chlamydophila pneumoniae	NC_002180.1	No alignment was found			No alignment was found		
Haemophilus influenzae	NZ_LN831035.1	No alignment was found			No alignment was found		
Legionella pneumophila	NZ_LR134380.1	No alignment was found			No alignment was found		
Mycobacterium tuberculosis	NC_000962.3	No alignment was found			No alignment was found		
Streptococcus pneumonia	NZ_LN831051.1	No alignment was found			No alignment was found		
Streptococcus pyogenes	NC_002737.2	No alignment was found			No alignment was found		
Bordetella pertussis	NC_018518.1	No alignment was found			No alignment was found		
Mycoplasma pneumoniae	NZ_CP010546.1	No alignment was found			No alignment was found		
Pneumocystis jirovecii (PJP)	GCF_001477535.1	No alignment was found			No alignment was found		
Candida albicans	GCF_000182965.3	No alignment was found			No alignment was found		
Pseudomonas aeruginosa	GCF_000006765.1	No alignment was found			No alignment was found		
Staphylococcus epidermis	GCF_000007645.1	No alignment was found			No alignment was found		
Staphylococcus salivarius	CP013216.1	No alignment was found			No alignment was found		
Staphylococcus aureus	NC_007795.1	No alignment was found			No alignment was found		
BtRf-AlphaCoV/HuB2013	KJ473818.1	No alignment was found			N/A	N/A	95.83 %
BtRf-AlphaCoV/JL2012	KJ473811.1	No alignment was found			N/A	N/A	95.83 %
Pangolin coronavirus MP789	MT084071.1	No alignment was found			N/A	N/A	95.83 %
Bat coronavirus RacCS203	MW251308.1	N/A	100%	N/A	N/A	N/A	100%
Bat coronavirus RaTG13	MN996532.2	N/A	N/A	96.15 %	N/A	N/A	100%
BetaCoV/Rm/Yunnan/YN02/2019	MZ081382.1	N/A	N/A	92.3 1%	N/A	N/A	100%
Bat SARS coronavirus HKU3	DQ022305.2	No alignment was found			100%	N/A	95.83 %
Bat coronavirus isolate B15-21	KU528591.1	No alignment was found			N/A	N/A	95.83 %
BtCoV/273/2005	DQ648856.1	No alignment was found			N/A	N/A	95.83 %

Bat SARS coronavirus Rf1	DQ412042.1	No alignment was found	N/A	N/A	95.83 %

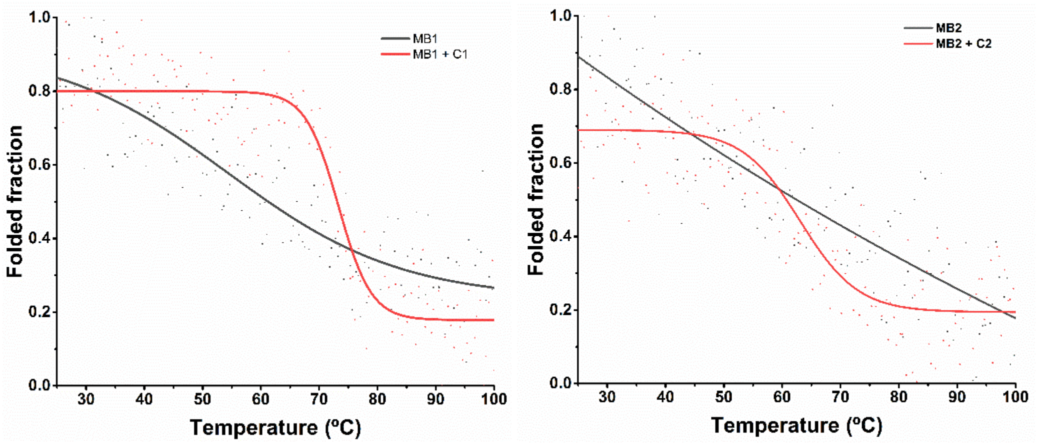

Figure S2 – Melting Curves Of MB1 and MB2 in presence os complementary sequences C1 and C2, respectively.

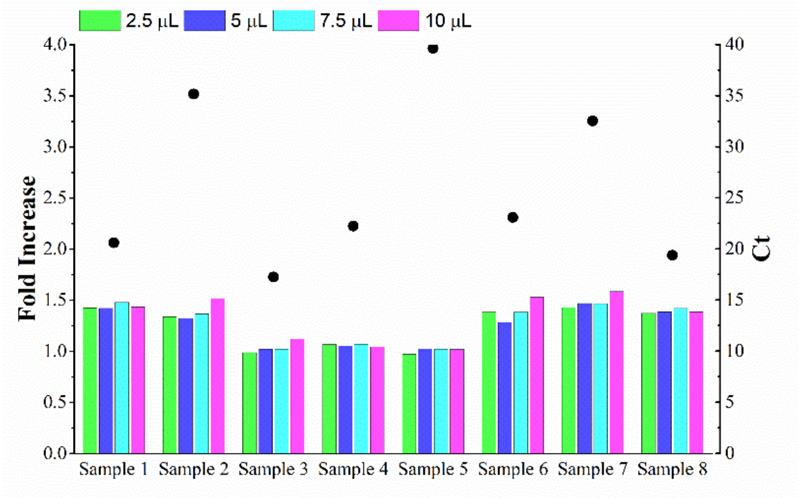

Figure S3 – Correlation between cycle threshold (Ct) amplification of SARS-CoV-2 in human samples, fluorescence fold-increase relative to the no template control (NTC) and samples with different amount of RNA viral.
